# Leveraging Cell‐Free Supernatants of Phyllospheric Bacteria to Combat Wheat Pathogens and Boost Growth

**DOI:** 10.1002/pei3.70063

**Published:** 2025-07-02

**Authors:** Noshaba Saleem, Ayesha Badar, Rhea Aqueel, Umer Zeeshan Ijaz, Kauser Abdulla Malik

**Affiliations:** ^1^ Kauser Abdulla Malik School of Life Sciences Forman Christian College (A Chartered University) Lahore Pakistan; ^2^ Water & Environment Research Group University of Glasgow, Mazumdar‐Shaw Advanced Research Centre Glasgow UK; ^3^ National University of Ireland Galway Ireland; ^4^ Department of Molecular and Clinical Cancer Medicine University of Liverpool Liverpool UK; ^5^ Pakistan Academy of Sciences Islamabad Pakistan

**Keywords:** *Blumeria graminis*, cell free supernatant, NMR, *Puccinia striiformis*, wheat

## Abstract

Wheat is a major staple crop in Pakistan but faces significant threats from biotic stressors such as powdery mildew (*Blumeria graminis*) and stripe rust (*Puccinia striiformis*). These fungal diseases can drastically reduce wheat yields, leading to substantial economic losses. This study aimed to evaluate the antifungal efficacy of Cell Free Supernatants (CFSs) from *Serratia marcescens* and *Fictibacillus* spp. (both originally derived from phyllosphere of *Gossypium arboreum)* against these fungal pathogens and assess their impact on wheat growth and yield. Wheat plants ( *Triticum aestivum* , Galaxy‐2013) were treated with CFSs from both strains, alongside control groups treated with Salicylic Acid (SA), Gibberellic Acid (GA3), and water (negative control). Disease severity was assessed using leaf area and spore count assays. Plant growth parameters, including height, fresh and dry weight, grain count, and spike number, were measured. Metabolomic analysis of the CFSs was conducted using ^1^H NMR. CFS treatments significantly reduced disease severity, with the lowest average percentage disease (APD) observed in the *Serratia marcescens* group for stripe rust (1.86%) and in the GA3 group for powdery mildew (1.30%). *Fictibacillus* spp. CFS also reduced the severity of both pathogens. CFS from *Serratia marcescens* resulted in the highest grain count and biomass. NMR analysis revealed the presence of antifungal and growth‐promoting compounds in the CFSs. CFSs from *Serratia marcescens* and *Fictibacillus* spp. show promise as effective bio‐stimulants and biocontrol agents against fungal diseases in wheat, offering a sustainable alternative to chemical treatments. Their application could improve wheat yield and contribute to more sustainable agricultural practices.

## 1 Introduction

The agriculture sector benefits from the country's rich endowment of natural resources, encompassing fertile arable land and ample water resources, which collectively constitute the bedrock of Pakistan's agricultural prosperity. Among the myriad crops cultivated in Pakistan, wheat is the most cultivated crop and serves as a staple food source (Ahmad 2009). Wheat contributes a substantial 10.3% to industry and an appreciable 1.8% to the nation's overall GDP (Imran et al. 2022). Despite the critical role of wheat in Pakistan's agriculture, numerous challenges threaten crop yields and quality. These challenges are categorized into biotic and abiotic factors (Malhotra 2014).

Biotic factors encompass diseases, insects, pests, and weeds, while abiotic factors include climate change and other environmental stressors (Braun et al. 2010). Among these challenges, powdery mildew, caused by the fungus *Blumeria graminis*, poses a significant threat to wheat production worldwide. This highly contagious plant disease affects wheat predominantly during its lush green vegetative stage, impacting the plant's photosynthetic rate, leaf assimilation index, respiration rate, and overall grain quality (Zhu 2017). Severe infections can even lead to plant death and substantial crop losses, with reductions of up to 40% in grain size and weight (Alam 2013). Globally, stripe rust inflicts an annual economic loss exceeding $5 billion in wheat production, with Pakistan being one of the hardest‐hit regions. These fungal infections, along with other biotic stresses, cause substantial damage to wheat crops, leading to yield losses that can range from 10% to 50% (Din 2023). Another prominent biotic stressor is stripe rust, caused by *Puccinia striiformis*, which can rapidly develop into severe epidemics, devastating crops under favorable weather conditions (Zhao 2016). Environmental factors, especially moisture and temperature, play a critical role in the spread of stripe rust. High moisture levels are essential for the rust pathogen's ability to infect host plants, with temperature ranges between 7°C and 12°C being particularly conducive to sporulation (Kiani 2021). Stripe rust (*Puccinia striiformis*) stands as the most dominant wheat disease in Asia, while powdery mildew (*Blumeria graminis*) ranks as the fourth most prevalent (Wellings et al. 2011). In Pakistan, stripe rust affects approximately 70% of the wheat cultivation area, contributing to significant agricultural challenges (Harmeet Singh Bakala 2022). It becomes, therefore, a necessity to devise potential strategies to counter these diseases in economically and environmentally resilient approaches.

Amidst these challenges, there is a growing interest in exploiting plant beneficial microbes, particularly Plant Growth‐Promoting Bacteria (PGPB), to enhance agricultural sustainability (Sun et al. 2024). Soil microbiota can improve soil productivity and assist plants in coping with various stressors. Understanding the intricate interactions between plants and microbes, using advanced multi‐omics approaches, has become essential for fostering sustainable agriculture (Putter 1981). PGPB are well known to enhance crop growth and resilience. However, challenges related to their formulation and persistence in soil need to be addressed. Researchers are exploring the use of cell‐free supernatants (CFSs) derived from bacterial cultures as bio‐stimulants and biocontrol agents (Rondina et al. 2020). C FSs contain various metabolites, such as phytohormones and chelating agents, which can positively impact plant growth and combat phytopathogenic fungi (Jones 2019). The stability of Cell‐Free Supernatant (CFS) applied as a soil drench or foliar spray, in comparison to directly applying Plant Growth‐Promoting Rhizobacteria (PGPR), often depends on factors such as formulation, storage conditions, and the specific microorganisms involved (Pellegrini et al. 2020). CFS can have improved stability as it protects the bioactive compounds from environmental stressors and degradation, potentially providing more consistent benefits to plants over time (Isabel et al. 2024).

This study is aimed at evaluating the antifungal activity of CFSs against *Blumeria graminis* and *Puccinia striiformis*, the causative agents of powdery mildew and stripe rust in plants, respectively. Additionally, an understanding of how CFSs affect wheat morphological characteristics was sought. Through metabolomic analysis using NMR technology, it was aimed to gain deeper insights into the composition and potential benefits of these CFSs of PGPBs for sustainable agriculture.

## 2 Materials and Methods

### 2.1 Bacterial Isolates Used in the Study

The strains selected for this study included *Serratia marcescens* and *Fictibacillus* spp., which were previously isolated from the phyllosphere of the Cotton Leaf Curl Virus (CLCuV)‐naturally‐tolerant cotton species, *Gossypium arboreum* , variety FDH‐228, by Aqueel et al. (2023). Table S1 lists the accession numbers along with isolation source and cell and colony morphology details.

### 2.2 Study Design

**FIGURE 1:**
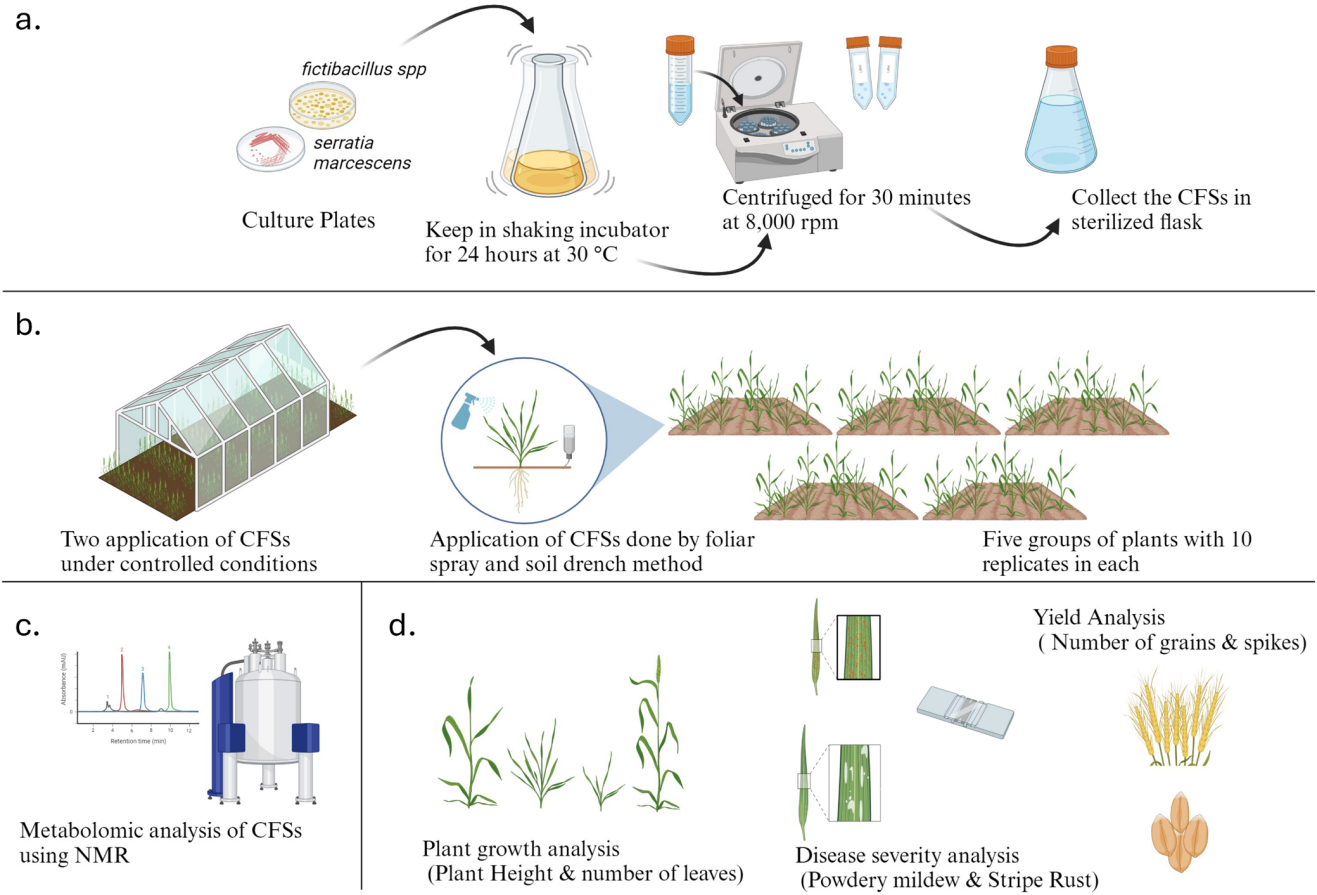
Illustrative diagram for the general workflow of the experiment (Biorender.com). (a) Cell‐Free Supernatant preparation, (b) Cell‐Free Supernatant application and fungal inoculation, (c) ^1^H NMR for identifying metabolites present in the CFSs, (d) Plant growth analysis and disease severity assessment.

Galaxy‐2013 variety of *Triticum aestivum* was chosen for carrying out the whole pot experiment for this study, owing to its general susceptibility to biotic stresses. The seeds for Galaxy‐2013 were sterilized and germinated on water agar until they became 2.5 cm‐long seedlings. The experiment was set in five application groups, namely, *Serratia marscens, Fictibacillus* spp., exogenous Salicylic Acid (SA), exogenous Gibberellic Acid (GA3), and Negative Control (NC). Each application group consisted of 10 pots or replicates. Each seedling was grown in a single 20 cm diameter pot, containing approximately 700 g of double‐sterilized nursery soil. The plants were kept in a climate‐controlled room with artificially set photoperiod for 14 days post germination. Two episodes of Cell Free Supernatants (CFSs), Salicylic Acid (SA) and Gibberellic Acid (GA3) were applied to the respective groups during this time. The Negative Control group received only the application of sterilized distilled water. The plants were moved to Net‐House I in Forman Christian College University (31.5222° N,74.3320° E) for fungal inoculation 21 days post germination (vegetative stage of growth, with beginning of tillering), which acted as a hub of fungal spores for *Blumeria graminis* and *Puccinia striiformis*. Here the plants were kept until maturity and disease severity assays were carried out. Test group application details and CFSs application regime are given in Table S2 and Figure S1, respectively. A schematic diagram showing the general layout of the experiment is depicted in Figure 1.

### 2.3 Cell Free Supernatant Preparation

The bacterial isolates *Serratia marcescens* and *Fictibacillus* spp. were cultured in sterilized half‐strength Tryptic Soy Broth (TSB) medium (Merck Millipore). After inoculation, incubation was carried out at 30°C for 24 h in a shaking incubator at 300 rpm in order to obtain 10^8^ cells per mL. Following incubation, OD of the visibly turbid bacterial culture was recorded at a wavelength 600 nm, and then they were centrifuged at 8000 rpm for 30 min at 4°C. The supernatants obtained were transferred to clean sterile falcon tubes. To ensure removal of bacterial cells, these CFSs were further filtered with a 0.2 um syringe filter. 100 μL of the prepared CFS aliquot was spread on half‐strength TSA plates and incubated at 30°C for 24 h in order to cross‐check the absence of any residual bacterial cells (Monjezi 2023).

### 2.4 Seed Preparation and Sowing Plan

Galaxy‐2013 wheat seeds were sterilized following the general protocol given by Lindsey 2017. Briefly, the seeds were washed with a 20% sodium hypochlorite solution for 1 to 2 min, followed by sterile distilled water, 70% ethanol (3–4 min), and sterile distilled water again. Sterilized seeds were air‐dried under a Level 2 laminar flow hood and stored in sterile falcon tubes.

Sterilized seeds were soaked briefly in autoclaved distilled water to initiate germination. Water agar plates were used for germination, maintaining optimal moisture levels and a controlled environment at 28°C in the dark for 7 days. Germination progress was monitored, and parameters including germination rate and microbial growth were recorded.

A sowing plan with 5 groups, each having 10 plant replicates, was implemented. Nursery soil was sieved, double autoclaved, dried, and placed in plastic pots. Sterilized wheat seeds were sown and moistened with autoclaved distilled water. The sowing was done according to the completely randomized block design (Figure S1) made using the agricolae package in R v4.4.3 (de Mendiburu 2023). Plants were grown in a controlled environment with an 8‐h dark and 16‐h light photoperiod for 14 days. Detailed records, including labeling, sowing date, Hoagland solution and CFSs application dates, and growth observations, were maintained.

### 2.5 Cell‐Free Supernatant Application

7 days after germination, the CFSs were applied to the seedlings through both the soil drench method and foliar spray to the leaves. The application quantity of 1 mL/g of soil was maintained. The second episode of CFSs application was given on the 14th day after germination. Equal volumes of the phytohormone solutions Salicylic Acid (0.4 mgmL^−1^) and Gibberellic Acid (0.05 mgmL^−1^) were also applied to the SA and GA3 plant groups, respectively, through the foliar spray method. The CFSs and phytohormone application regime is given in Figure S2.

### 2.6 Fungal Inoculation

Wheat plants of 14 days of age were moved to the Forman Christian College University's Net House I, for fungal inoculation. To ensure uniform fungal spore inoculation (*Blumeria graminis* and *Puccinia striiformis*), heavily infected wheat plant leaves showing severe symptoms (Figure S3) of the diseases were obtained from an open field on Multan Road, Lahore, Pakistan (N 31°30′ 21.6468″, E 74°20′ 38.4684″) and crushed in sterile 1× T.E. Buffer. Spores were quantified using a hemocytometer and rubbed onto test and control plant leaves in equal volume using a sterile cotton bud. Separate fungal spore suspensions were prepared for each fungal pathogen. All plants, regardless of the application group, were inoculated with spore suspensions of both *Blumeria graminis* and *Puccinia striiformis*.

### 2.7 Disease Incidence Assays

#### 2.7.1 Diseased Leaf Area Assay

Disease assessment involved using a standardized scale for symptom severity, both qualitatively (presence/absence) and quantitatively (rating on a 0–10 scale). Healthy and diseased leaf area was measured for all plants across groups, 39 days post fungal inoculation (60th day post germination), when the plants had entered their flowering (anthesis) stage. The following equation was used to calculate the disease severity index (Bedika 2016).

$$\mathrm{DI}=\frac{\sum \left(\text{Number of plants}\times \text{Number of degree in symptoms}\right)}{\text{Total number of plants}\times 5\;\left(\text{Maximum degree in symptoms}\right)}\times 100$$

$$\mathrm{DSI}=\frac{\text{Percentage Disease Severity}\;}{100\;}\times \text{Total Score}$$

For calculating the Average Percentage Disease (APD), the following formula was applied (Venkata 2013).

$$\mathrm{APD}=\frac{\mathrm{Sum}\;\text{of grades}}{\text{Total number of leaves analyzed}\times \text{maximum disease grades}\;}\times 100$$

Table S3 summarizes the powdery mildew disease severity grades, ranging from mild to severe symptoms. The maximum grade on a 0–9 scale was nine.

#### 2.7.2 Spore Count Assay

The spore count assay was performed individually for the plant pathogens powdery mildew (*Blumeria graminis f*. sp. *tritici*) and Stripe Rust (*Puccinia striiformis f*. sp. *tritici*). For this, a separate experiment was set, containing the same five application groups, with 5 plants or replicates in each group. These application groups underwent identical CFSs, SA, and GA3 applications as described previously. The destructive sampling for this assay was done 39 days post fungal inoculation (60th day post germination), when the plants had entered their flowering (anthesis) stage. Equal amounts of leaf samples from all five experimental groups were collected, ensuring they displayed visible disease symptoms. These samples were washed to remove surface dust. Spore suspensions were prepared by crushing infected leaf tissues with sterile 1× T.E. buffer. Hemocytometers were used for spore counting, with recommended concentrations of around 2.5 × 10^5^ to 2.5 × 10^6^ spores/mL. A cover slip was placed on the glass slide and spores were observed under a microscope at 400× magnification. Spores were counted in multiple randomly selected squares of the counting chambers. The following equation given by (Schütz 2020) was used to calculate the spore concentration per unit volume.

$$\text{Total spore Count}=\frac{\text{Number of spores}\;\times \text{Dilution Fector}.10^{4}}{\text{Number of squares counted}\;\mathrm{on}\;\text{hemocytometer}}$$

### 2.8 Molecular Identification of *Blumeria Graminis* Using Species‐Specific Primers

Total DNA was extracted from diseased plant leaves using the CTAB method (Aboul‐Maaty and Oraby 2019). The details of DNA extraction are given in Appendix S1. Amplification of the ITS2 region of the 18S rRNA gene was conducted with forward ITS primer (5′‐TAGAGGAAGTAAAAGTCGTAA‐3′) and reverse ITS primer (5′‐TTYRCTRCGTTCTTCATC‐3′) (Toju et al. 2012). The PCR reaction profile is given in Table S4. The PCR profile for amplifying the 18S rRNA ITS1‐ITS2 region involves initial denaturation at 95°C for 5 min, followed by annealing at 58°C for 1 min, cycled 40 times. Extension is at 72°C for 1 min, and there is a final extension at 72°C for 10 min before holding at 4°C. PCR products were visualized as 600 bp bands on a 1.5% agarose gel after electrophoresis.

For molecular identification of *Blumeria graminis*, the species‐specific primers LidBg21/22 for the CYP51 gene, developed by Kuzdralinski et al. (2018), were used (LidBg21: 5′‐AATTCGGCTTTAGCATTGCGTT‐3′, LidBg225′‐TTCGTGTTCCCCAGAATATATCA‐3′). The recipe for the PCR mixture is given in Table S5. The PCR profile for the amplification of the CYP51 gene consists of an initial denaturation phase at 95°C for 5 min, carried out only once. This is followed by 40 cycles of denaturation at 95°C for 1 min, annealing at 62°C for 45 s, and extension at 72°C for 1 min. The final extension is conducted at 72°C for 10 min. The PCR products were visualized on a 2.5% agarose gel as a band size of 200–250 bp.

### 2.9 Plant Growth and Yield Analysis

Plant growth analysis encompassed various parameters. Phenotypic characteristics such as plant height and leaf count were recorded every 3 days. Plant height was measured using a measuring tape placed parallel to the plant stalk. The wheat crop was harvested by hand on the 80th day after germination (DAG) with minimal damage to the plants. Spikes were counted and collected separately. Yield parameters like fresh and dry weight and grain count were measured. The fresh weight of shoots was recorded post‐harvest using an electronic weighing balance (Metra BSM Analytical Balances_BSM‐220‐4). The dry weight for both roots and shoots was obtained after 24 h of drying at 60°C. Root analysis involved staining the roots with a 1% w/v methylene blue solution, scanning with an Epson scanner Expression 1000XL, and analysis using WinRhizo software, enabling non‐invasive measurement of root characteristics like area and length.

### 2.10 Statistical Analysis

IBM SPSS Statistics 25 software was employed for statistical analysis for data related to growth factors, disease severity assays, and yield analysis. The significant difference between test groups was calculated using one‐way univariate analyses of variance (One‐Way ANOVA test) and Tukey's honestly significant difference (HSD) *post hoc* test in IBM SPSS Statistics. The differences among test groups were considered significant at *p*‐value less than 0.05 with significance values as: *p < 0.05, **p < 0.01, or ***p < 0.001.

### 2.11 Antifungal Characterization of CFSs on Field Soil Samples

The fungal Colony Forming Units per mL (CFU/mL) were assessed for soil samples applied with CFSs. Soil was collected from both the field and the net house locations at Forman Christian College University (31.5222° N,74.3320° E) with six random samples from each location. The samples were labeled, and replicates were thoroughly mixed after removing debris. The soil samples were transferred to sterile and dried petri plates, and CFSs from both bacterial strains were applied in three episodes and incubated at 28°C. Serial dilutions of treated soil were prepared, spread on Potato Dextrose Agar plates, and incubated for 6 days at 28°C. The distinct, well‐defined fungal colonies on each plate were then counted to determine fungal load based on CFU/mL. The CFU/mL of the soil samples was calculated using the following equation (dos Santos 2020):

$$\mathrm{CFU}/\mathrm{mL}=\frac{\text{Number of Colonies Counted}\times \text{Dilution Factor}\;}{\text{Volume Plated in}\;\mathrm{mL}}$$

### 2.12 Metabolomic Analysis of CFSs Using ^1^H NMR

The prepared CFS for *Fictibacillus* spp. was sent for D1 ^1^H Proton NMR to Lahore University of Management Sciences, Syed Babar Ali‐School of Science and Engineering, Lahore. The samples were run on the Bruker Avance Neo, 600 MHz instrument. The probe used was TXI and the solvent was CDCl_3_. The baseline correction and phase correction for the ^1^H NMR raw data was done in the software MestReNova, version 14.3.2, by MestReLab Research Inc., followed by peak picking. This highlighted all the peaks obtained in the spectrum. The peak table was then exported for downstream analysis.

The compounds were identified using the Automated Spectral Processing System for NMR, AlpsNMR, package (https://bioconductor.org/packages/release/bioc/html/AlpsNMR.html) on R (version 4.3.0). The sample chemical shift table in the .csv format was loaded into RStudio, and compounds were identified using the reference NMR compounds chemical shifts database from the Biological Magnetic Resonance Data Bank (BMRB) (https://bmrb.io/ref_info/csstats.php?restype=nstd&set=full). The AlpsNMR package cross‐referred to all the chemical shifts in the sample and gave the relative compound associated with the shift and intensity. The identified compounds table was saved in .csv file format using the “write.csv” command.

## 3 Results

### 3.1 The Potential of Cell Free Supernatants From Phyllospheric Bacteria in Powdery Mildew and Stripe Rust Suppression

The disease incidence assays carried out on wheat plants demonstrated strongly the powdery mildew and stripe rust disease suppression through the application of the CFSs of *Serratia marcescens* and *Fictibacillus* spp. The disease percentage data obtained after 80 days of pot analysis highlight the effectiveness of various treatments in reducing the severity of the two diseases (Data S1).

**FIGURE 2:**
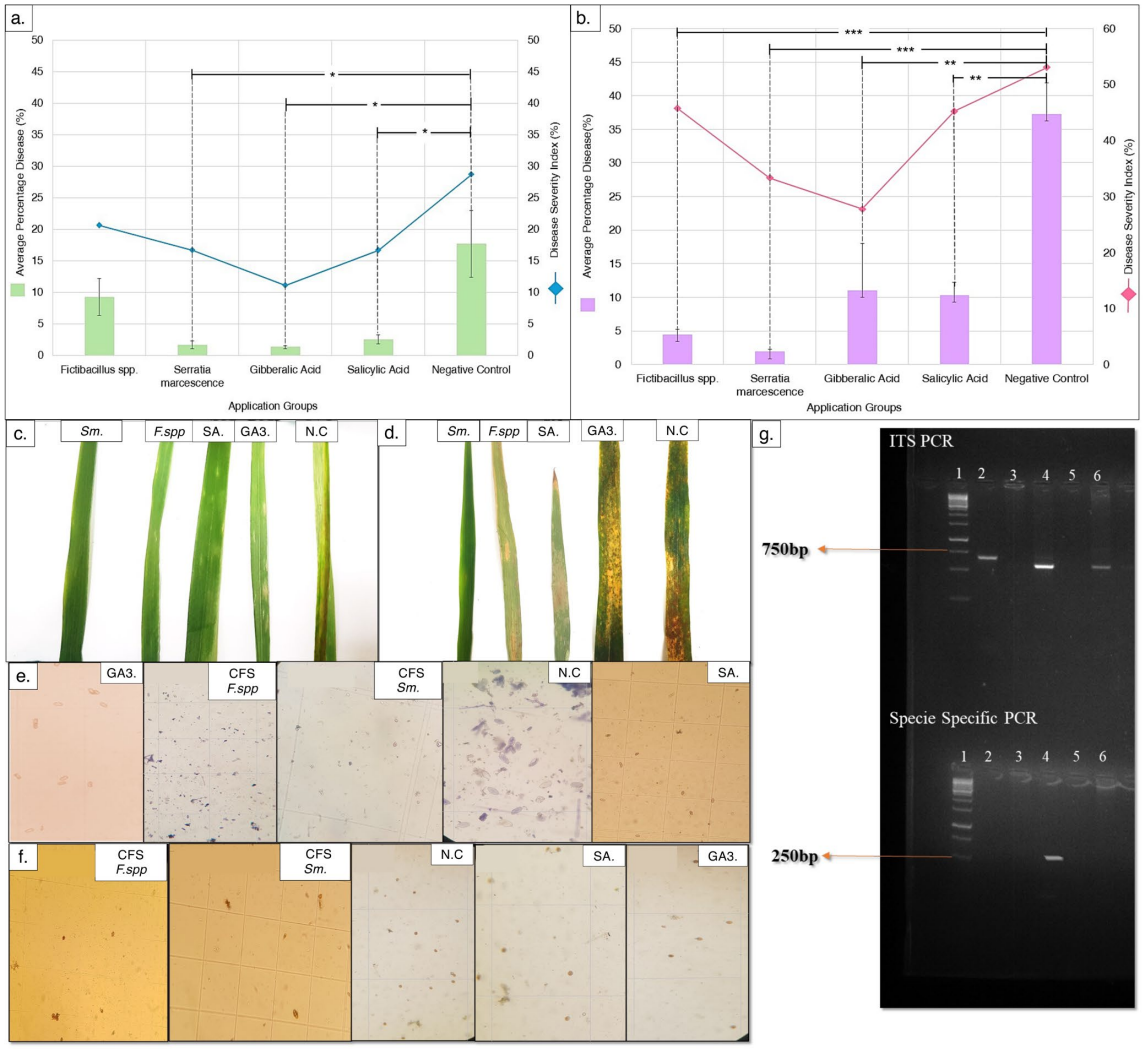
Disease‐severity assay based on diseased leaf area assessment. (a) Average Percentage Disease for *Blumeria graminis* in all application groups, (b) Average Percentage Disease for *Puccinia striiformis* in all application groups, (c) *Blumeria graminis* symptoms as seen on plant leaves from all groups. The differences among test groups were considered significant at *p*‐value less than 0.05 with significance values as: *p < 0.05, **p < 0.01, or ***p < 0.001. (d) *Puccinia striiformis* symptoms as seen on plant leaves from all groups, (e) *Blumeria graminis* spores as seen under light microscope, (f) *Puccinia striiformis* spores as seen under light microscope, (g) PCR amplification of fungal ITS‐2 gene (top) and *Blumeria graminis* confirmation using species specific primers (bottom); Lane 1:1 kb ladder, Lane 2: Group applied with CFS of *Fictibacillus* spp., Lane 3: Group applied with CFS of *Serratia marcescens* , Lane 4: NC, Lane 5: SA, Lane 6: GA3.

For powdery mildew, the Average Percentage Disease (APD) and Percentage Disease Index (PDI) were lowest in the Gibberellic Acid treatment group, with values of 1.30% and 11.11, respectively, indicating strong disease suppression. The CFS of *Serratia marcescens* and Salicylic Acid also showed low APD values of 1.62% and 2.49%, respectively. The *Fictibacillus* spp. group had an APD of 9.22%, showing moderate effectiveness, while the Negative Control group exhibited the highest APD (17.65%) and PDI (28.70), indicating that disease was most severe without treatment (Figure 2a).

For stripe rust, the treatment with CFS of *Serratia marcescens* showed the lowest APD (1.86%), suggesting effective disease suppression. Gibberellic Acid showed an APD of 10.98%, indicating less effectiveness compared to other treatments. Salicylic Acid and *Fictibacillus* spp. had similar PDIs around 45, but *Fictibacillus* spp. had a lower APD of 4.39%, suggesting it was more effective than Salicylic Acid. Again, the Negative Control group showed the highest APD (37.25%) and PDI (53.06), indicating the highest disease severity without treatment (Figure 2b). Figure 2c,d depict the disease symptoms for *Blumeria graminis* and *Puccinia striiformis*, respectively, as they appear on the leaves from each group.

**FIGURE 3:**
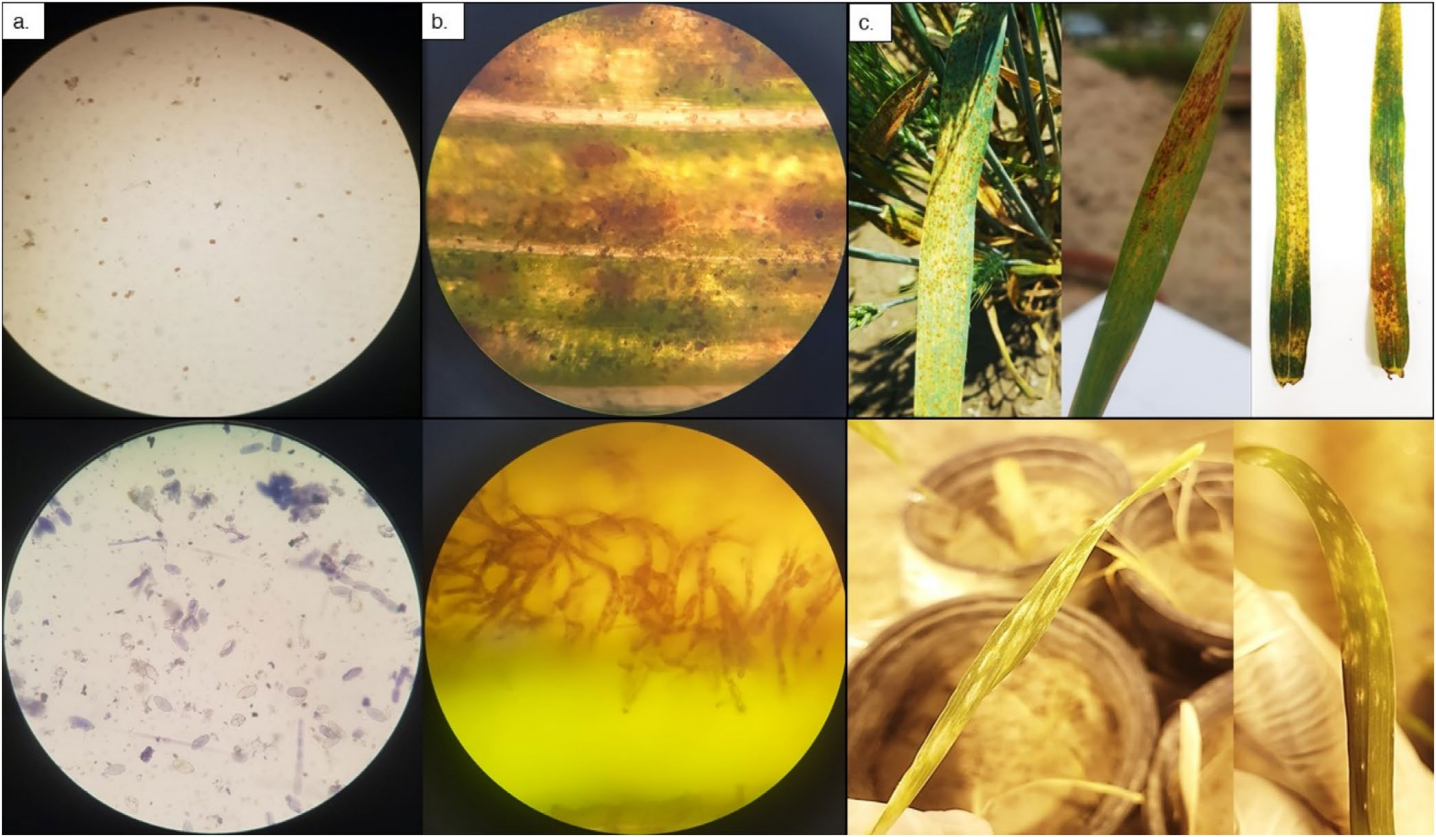
Stripe rust (*Puccinia striiformis*) and powdery mildew (*Blumeria graminis*) disease severity in negative control plant leaves in absence of any biocontrol application, (a) Top: Spores of *Puccinia striiformis* seen under light microscope, Bottom: Spores of *Blumeria graminis* seen under light microscope, (b) Top: Leaf infected with *Puccinia striiformis* seen under light microscope, Bottom: Leaf infected with *Blumeria graminis* seen under light microscope, (c) Top: Severe symptoms of *Puccinia striiformis* seen with naked eye on control plant leaves, Bottom: Severe symptoms of *Blumeria graminis* seen with naked eye on control plant leaves.

The disease incidence for powdery mildew and stripe rust was also recorded using the spore count technique. Figure 2e depicts the characteristic spores of *Puccinia striiformis* under the light microscope for all application groups, whereas those for *Blumeria graminis* can be seen in Figure 2f. The molecular presence of *Blumeria graminis* is shown in the PCR‐based species‐specific gene amplification (Figure 2g). The comparative symptoms on the control plants can be seen in Figure 3 for both *Blumeria graminis* and *Puccinia striiformis*.

**FIGURE 4:**
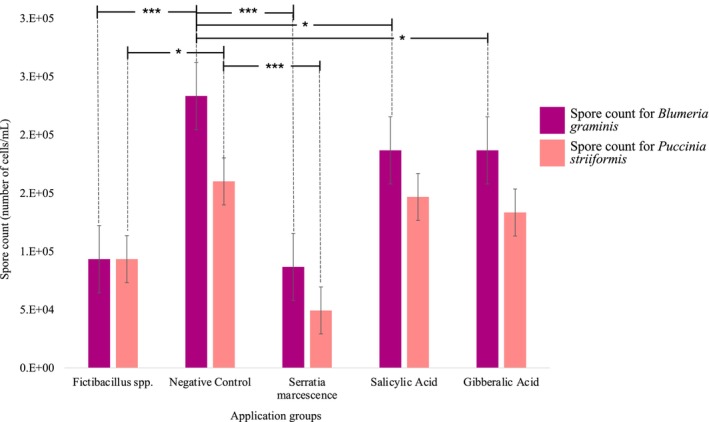
Disease severity assay based on spore count for both *Blumeria graminis* and *Puccinia striiformis*. The differences among test groups were considered significant at *p* ‐ value less than 0.05 with significance values as: *p < 0.05, **p < 0.01, or ***p < 0.001.

The *Fictibacillus* spp. CFS treatment group exhibited the lowest spore counts for both pathogens, with *Blumeria graminis* at approximately 1 × 10^5^ cells/mL and *Puccinia striiformis* at a similar level. On the other hand, the Negative Control group shows the highest spore counts, with *Blumeria graminis* approaching 3 × 10^5^ cells/mL and *Puccinia striiformis* around 2.5 × 10^5^ cells/mL (Figure 4).

### 3.2 Effect of CFSs From Phyllospheric Bacteria on Plant Growth Enhancement

**FIGURE 5:**
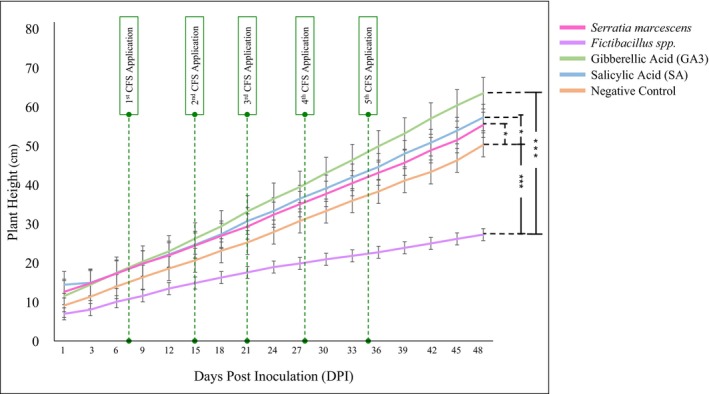
Plant height progress after fungal inoculation in all experimental groups. Changes have been in the caption text: The differences among test groups were considered significant at *p*‐value less than 0.05 with significance values as: *p < 0.05, **p < 0.01, or ***p < 0.001.

Several phenotypic characteristics for growth assessment of the test and control plants were studied (Data S2). For plant height, which was observed post‐fungal infection, Gibberellic Acid treatment was the most effective in promoting plant growth, starting with an average height of 4.59 in. on Day 1 and reaching 25.05 in. by Day 48. Similarly, plants treated with Salicylic Acid also exhibited significant growth, increasing from 6.14 in. on Day 1 to 22.61 in. by Day 48. In comparison, the CFSs of *Serratia marcescens* and *Fictibacillus* spp. treatments led to moderate growth, with the CFS of *Serratia marcescens* plants growing from 5.02 in. to 21.86 in. The Negative Control group, which did not receive any growth‐promoting treatment, exhibited the least growth, starting at 3.64 in. and reaching 19.84 in. by Day 48 (Figure 5).

**FIGURE 6:**
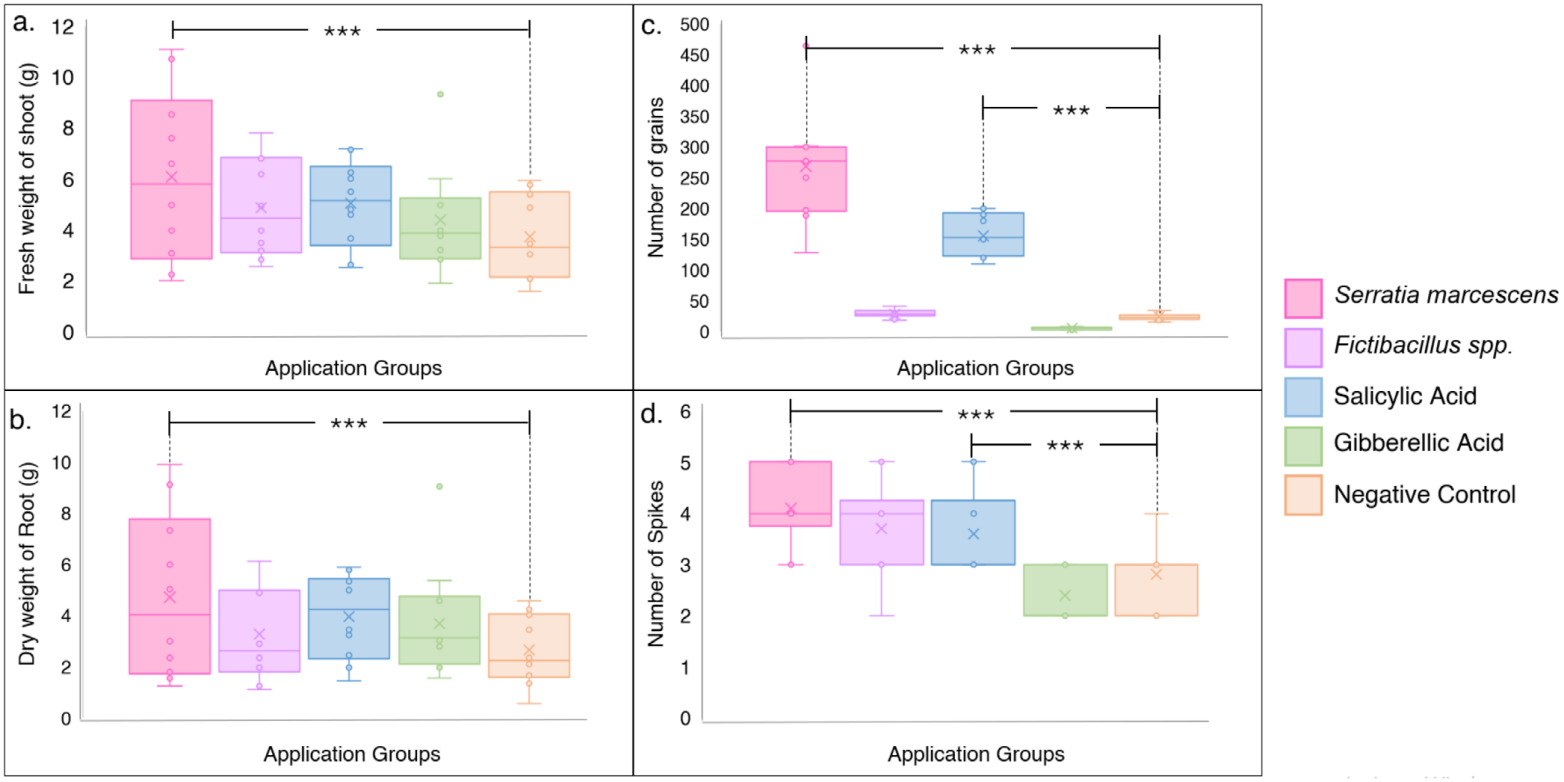
Measurement of plant phenotypic characteristics and yield for assessment of PGP by CFSs and phytohormones, (a) Shoot fresh weight in g, (b) Root dry weight in g, (c) Number of grains for yield assessment, (d) Number of spikes for yield assessment.

Plant growth was assessed based on several other growth factors as well, such as shoot fresh weight root dry weight, number of grains, and number of spikes. For shoot fresh weight, the CFS of *Serratia marcescens* treatment group shows the highest average value of 6.084 g. The CFS of *Fictibacillus* spp. and Salicylic Acid treatment groups also demonstrated high fresh weight averages (4.87 and 5.04 g, respectively). Gibberellic acid application and the Negative Control group had lower averages (4.40 and 3.75 g), suggesting that these treatments were less effective in promoting fresh biomass (Figure 6a). For root dry weight, the CFS of *Serratia marcescens* application group again showed the highest average dry weight of 4.78 g, followed by *Fictibacillus* spp. CFS treatment group (3.26 g) and Salicylic Acid treatment group (3.97 g). Gibberellic acid treatment group showed a moderate dry weight average of 3.69 g, while the Negative Control group had the lowest average dry weight at 2.64 g (Figure 6b).

For the number of grains, *Serratia marcescens* CFS application group significantly outperformed other treatments, with an average of 268.8 grains per plant. Salicylic Acid application group also showed a relatively high average number of grains (155.4), whereas *Fictibacillus* spp. CFS and Gibberellic acid application group, along with the Negative Control group, had much lower averages (29.5, 5.7, and 23.8 respectively), suggesting that these treatments were less effective in increasing grain production (Figure 6c). In terms of the number of spikes per plant, all treatments showed a narrow range of averages, with *Serratia marcescens* CFS application group leading at 4.1 spikes, closely followed by *Fictibacillus* spp. CFS group (3.7 spikes) and Salicylic Acid (3.6 spikes). Gibberellic acid application group and the Negative Control group had the lowest averages (2.4 and 2.8 spikes, respectively), suggesting that these treatments were less effective in promoting spike formation (Figure 6d). The plant group applied with CFS of *Serratia marcescens* consistently showed the best results across all measured parameters, particularly in fresh and dry weight, as well as grain production, indicating it may be a potent treatment for promoting overall plant growth and yield.

### 3.3 ^1^H NMR‐Based Metabolomic Analysis of CFSs for Plant Disease Management

**FIGURE 7:**
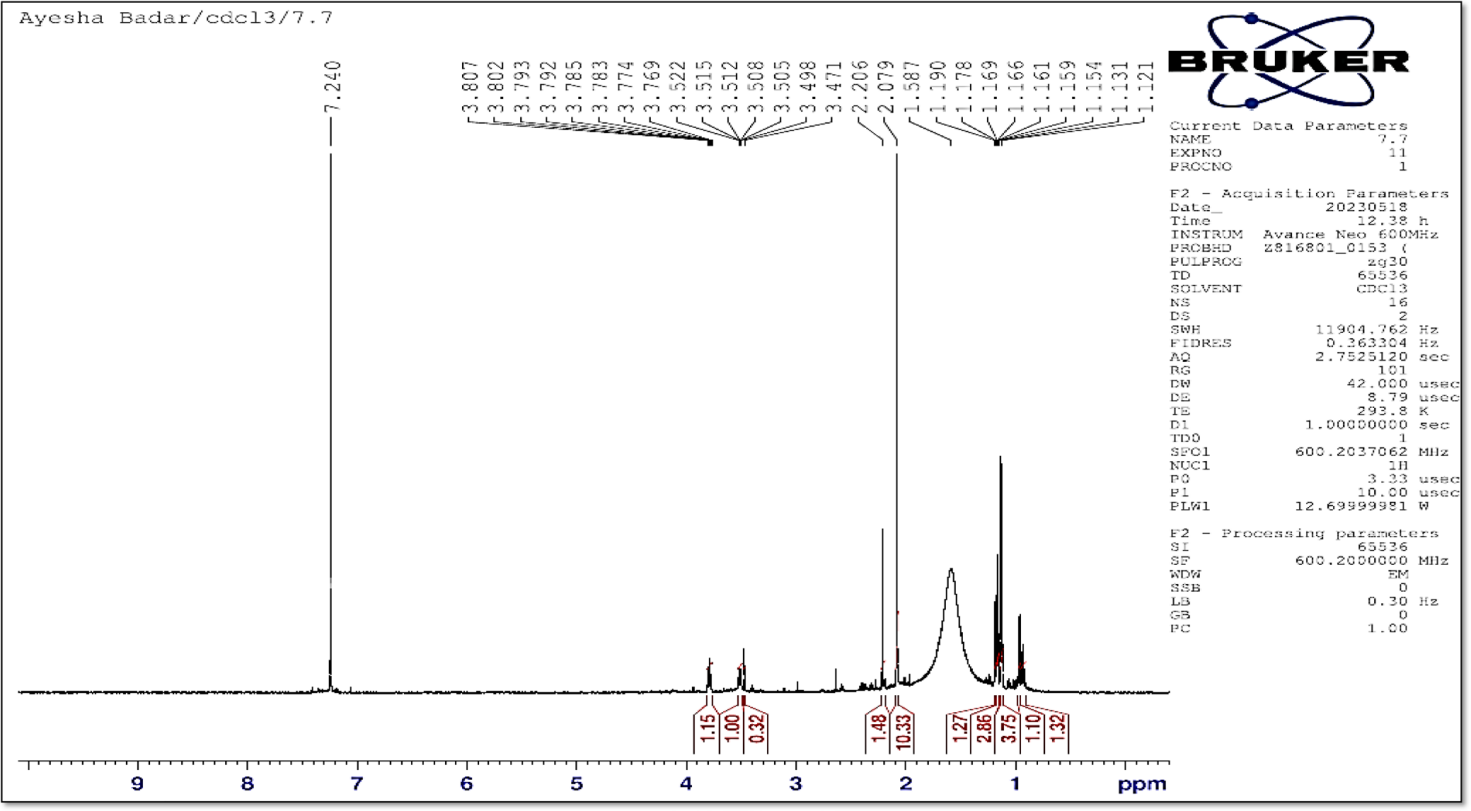
NMR spectrum of the CFS of Fictibacillus spp. obtained on a Bruker Avance Neo 600 MHz spectrometer, showing distinct chemical shifts and multiplicities.

The analysis of the CFS from the strain *Fictibacillus* spp. revealed a spectrum with chemical shifts ranging from 1.11 ppm to 7.61 ppm (Figure 7), indicating a variety of organic compounds present in the sample. The most intense peak, with an intensity of 1,279,953, was observed at a chemical shift of 2.28 ppm, suggesting a significant concentration of a particular compound at this chemical environment. Conversely, the lowest intensity recorded was 1480.5 at a chemical shift of 7.54 ppm. The compounds identified through the spectrum, compared against the BMRB database, included Tryptophanol, beta‐D‐xylopyranose (Obi 2022), Propane (Currie et al. 2022), etc., which are known for their antifungal, antibacterial, and plant growth‐promoting properties. This supports the earlier findings of the disease‐resistant characteristics of the CFS from the bacterial isolate of *Fictibacillus* spp. The detailed list of compounds with intensities above 50,000 is provided in Table 1, with further information on their properties corroborating the bioactive potential of the CFS. Data S3 shows a list of all the compounds and their relative IDs identified through the BMRB database.

**TABLE 1:** Compounds having relative intensities higher than 50,000, along with compound names and derived from BMRB database and relative descriptions.

Chemical shift (ppm)	Intensity	Compound Id	Atom Id	Compound name	Compound‐description
7.44	653,858	NCX	HH72	1‐(2‐DEOXY‐5‐O‐PHOSPHONO‐BETA‐D‐ERYTHRO‐PENTOFURANOSYL)‐5‐NITRO‐1H‐INDOLE‐3‐CARBOXAMIDE	Antibacterial and antifungal agents and a metabolite of dietary l‐tryptophan which is synthesized by human gastrointestinal bacteria
7.44	653,858	TPL	HE3	TRYPTOPHANOL	Tryptophan is an α‐amino acid that is used in the biosynthesis of proteins
2.4	112296.7	L2H	HN3	bis(4‐aminobutyl)dimethyl‐hexaoxa‐octaazaheptacyclo‐hexatriaconta‐dodecaene‐dione	It is an enoate ester, a methyl ester and a diester. It is functionally related to a methanol and fumaric acid.
2.28	1,279,953	XYP	H5B2	beta‐D‐xylopyranose	Xylose or wood sugar is an aldopentose, a monosaccharide containing five carbon atoms and an aldehyde functional group. (Obi 2022)
1.78	86023.3	HEM_ox	3HBC	PROTOPORPHYRIN IXCONTAINING FE	2 propionic and 2 vinyl side chains that is a metabolic precursor for hemes, cytochrome c and chlorophyll

## 4 Discussion

### 4.1 Cell‐Free Supernatants From Bacteria: A Multifaceted Approach to Sustainable Agriculture

Cell‐free supernatants (CFSs), the liquid fractions derived from bacterial cell cultures, are increasingly recognized for their multifaceted applications in various industries, particularly in agriculture. These CFSs are rich in bioactive molecules, offering notable advantages over traditional cell culture methods, including cost‐effectiveness and simplicity (Chen 2019). The growing interest in CFSs is driven by their potential to streamline manufacturing processes, reduce costs, and bypass the complexities associated with cell culture procedures. They are invaluable for studying the effects of secreted bioactive compounds on cells, crops, or organisms, and are particularly rich in enzymes, antimicrobial substances, signaling molecules, and metabolites (Mani‐López et al. 2022). These bioactive compounds within CFSs can be extracted, purified, and applied across various biotechnological fields, including pharmaceuticals, cosmetics, and biofertilizer production. The use of CFSs presents exciting opportunities for scientific advancements, particularly in agriculture and industrial innovation, by providing an efficient and versatile method for discovering and harnessing bioactive chemicals. This versatility opens up numerous avenues for research and development in the biological sciences and agricultural biotechnology (El‐Tanahy et al. 2024).

Cell‐free supernatants (CFSs), derived from bacterial cell cultures, are gaining attention for their biotechnological applications, particularly in sustainable agriculture. These supernatants contain bioactive compounds that influence plant growth, enhance disease resistance, and reduce dependence on chemical pesticides (Singh et al. 2025). The findings of this study demonstrate that CFSs from *Serratia marcescens* and *Fictibacillus* spp. significantly suppress powdery mildew (*Blumeria graminis*) and stripe rust (*Puccinia striiformis*) while also promoting wheat growth and yield. These results reinforce the potential of CFSs as effective biocontrol agents, offering an eco‐friendly alternative to synthetic fungicides (Bano et al. 2022). Metabolomic analysis of CFSs using ^1^H NMR confirmed the presence of bioactive metabolites with antifungal and plant‐growth‐promoting properties. Identified compounds such as tryptophanol, β‐D‐xylopyranose, and protoporphyrin IX containing iron suggest roles in disease suppression, nutrient uptake, and plant stress resilience. These findings align with previous studies that highlight the ability of bacterial metabolites to modulate plant physiological responses, reinforcing the significance of CFSs in integrated disease management strategies (Chen et al. 2024).

In the CFSs of plant growth‐promoting bacterial (PGPB) strains, a variety of metabolites have already been reported (Meena et al. 2020), varying according to the specific strain and environmental conditions. Although there is a diverse array of metabolites reported, noteworthy metabolites in PGPB‐CFSs include indole‐3‐acetic acid (IAA), a phytohormone that promotes root growth and overall plant development; siderophores, iron‐chelating compounds that enhance iron uptake, thereby aiding nutrient acquisition in plants; and volatile organic compounds (VOCs) such as hydrogen cyanide (HCN), an antibacterial compound that inhibits the growth of plant pathogens and protects against diseases. These metabolites contribute significantly to improved plant growth, efficient nutrient utilization, disease control, and overall plant health, making them integral to sustainable agriculture and increased crop productivity (Sarwar et al. 2018; Ventorino et al. 2019). Recent multi‐omic studies, such as those by Dow et al. (2023), have highlighted the extensive range of specialized metabolites produced by *Streptomyces* species, underscoring their potential in phytopathogen control and plant growth promotion.

Along with the reported benefits of the CFSs, their application to plants for growth promotion offers significant advantages over live bacterial cell application. Due to the absence of live bacterial cells, the concerns related to specificity and colonization, or constraints of cross‐species application, are eliminated. Without the need for bacteria to colonize the plant's rhizosphere, the beneficial compounds present in the CFSs, such as signaling molecules, enzymes, and antimicrobial substances, can directly influence plant growth and stress resilience. This approach bypasses potential issues like the need for bacteria to adapt to specific plant hosts or environmental conditions, making it a more universally applicable strategy across different plant species (Bhattacharyya and Jha 2012). Additionally, the use of CFSs reduces the risk of unintended ecological impacts, such as the introduction of non‐native bacterial strains, while still harnessing the growth‐promoting and disease‐suppressing properties of plant growth‐promoting bacteria (PGPB) (Sansinenea 2019).

### 4.2 Metabolomic Insights Into the Antifungal and Plant Growth Promoting Properties of CFSs of Phyllospheric *Serratia marcescens* and *Fictibacillus* spp.

In this study, the cell‐free supernatants (CFSs) derived from two bacterial strains were evaluated for their capacity to enhance biotic stress tolerance in wheat. The results demonstrated significant positive effects, including increased plant height, enhanced root dry weights, and overall biomass content when compared to control groups. Additionally, the CFSs effectively mitigated powdery mildew and stripe rust infections, evidenced by reduced disease incidence and lower spore counts. The impact of these CFSs on plant growth, disease resistance, and yield aligns well with previous research, underscoring their potential in agricultural applications. The PGPB strain *Serratia marcescens* significantly influenced plant biomass, height, and disease resistance, suggesting that CFS‐based biocontrol methods could serve as viable strategies for managing such lethal wheat diseases as the powdery mildew or the stripe rust. Plant height is a critical agronomic trait in wheat, influencing lodging resistance, yield potential, disease susceptibility, and stress tolerance (Khobra et al. 2019). Notably, our treatments did not result in excessive height increases that could compromise plant stability or overall productivity.

Metabolomic analysis of CFSs, utilizing techniques such as Nuclear Magnetic Resonance (NMR) spectroscopy, plays a crucial role in identifying and quantifying the metabolites present in these supernatants. This approach provides valuable insights into the metabolic profiles of bacterial supernatants and helps identify potential bioactive compounds responsible for promoting plant growth, enhancing disease resistance, and improving crop yields. While this approach provides an efficient and automated method for compound identification, it also carries certain limitations. The accuracy of spectral assignments depends on the quality of the reference database (BMRB), and some compounds with overlapping chemical shifts may not be distinctly identified. Additionally, the use of CDCl_3_ as a solvent may limit the detection of highly polar metabolites. The AlpsNMR package, while robust, does not confirm structural identities, necessitating further validation through orthogonal techniques such as high‐resolution mass spectrometry (HRMS) or spiking with authentic standards (Aretz and Meierhofer 2016).

The compound having the highest intensity in the ^1^H‐NMR spectrum of the CFS of *Fictibacillus* spp. included 1‐(2‐deoxy‐5‐O‐phosphono‐β‐D‐erythro‐pentofuranosyl)‐5‐nitro‐1H‐indole‐3‐carboxamide, which is a modified nucleoside analog, exhibiting potential roles in agricultural and biochemical research. This molecule is derived from indole‐3‐carboxamide, a known structure with biological activity, and it incorporates a 2‐deoxy‐pentofuranosyl moiety along with a phosphono group. The addition of these functional groups alters its interaction with biological systems. This compound is of particular interest due to its potential as a growth regulator in plants. Nucleoside analogs like this one can impact nucleic acid synthesis and cell signaling pathways, which are crucial for plant growth and development. The phosphono group can mimic phosphate groups in biological systems, potentially interfering with or modifying signaling pathways related to plant stress responses or growth. Additionally, the nitro group might contribute to oxidative stress mechanisms or act as a signaling molecule. In agriculture, such compounds could be used to enhance crop yield and stress resistance by modulating plant metabolic pathways or improving disease resistance. While specific studies on this exact compound might be limited, similar nucleoside analogs have been explored for their ability to affect plant growth and resilience to environmental stresses (Buchmann 2008).

Tryptophanol, another compound corresponding to a chemical shift in the ^1^H‐NMR spectrum in the CFS of *Fictibacillus* spp., is a naturally occurring compound related to tryptophan, an essential amino acid. Known for its role as a plant growth regulator, tryptophanol influences various physiological processes in plants by modulating auxin levels, which are crucial for cell elongation, root development, and overall plant growth (Ferrandi 2023). Tryptophanol has been explored for its potential to enhance plant growth, improve stress tolerance, and increase crop yields. By affecting auxin levels, tryptophanol promotes root development and improves nutrient and water uptake, which can be particularly beneficial under suboptimal conditions (Zhao and Kang 2016). Recent research highlights its role in advancing agricultural practices and enhancing crop productivity (El‐Tanahy et al. 2024).

β‐D‐Xylopyranose is a monosaccharide sugar that is a derivative of xylose, a five‐carbon sugar. In its β‐D‐pyranose form, it adopts a six‐membered ring structure that includes five carbon atoms and one oxygen atom, characteristic of pyranose sugars. β‐D‐Xylopyranose is important in various biological and industrial contexts. In plants, xylose is a component of hemicellulose, a major polysaccharide in the cell wall, contributing to the structural integrity of plant tissues. Understanding the role of xylose and its derivatives can be crucial for improving plant cell wall composition and plant growth. In agriculture, bacteria producing β‐D‐xylopyranose could be used to improve soil health or enhance plant growth. The production of xylose by soil bacteria might influence the availability of carbohydrates in the soil, which can affect plant‐microbe interactions and overall plant health (Xu et al. 2019). Additionally, understanding how these bacteria produce and utilize β‐D‐xylopyranose can lead to innovative strategies for modifying plant cell walls, potentially improving crop resilience and yield.

Protoporphyrin IX containing iron plays a crucial role in plant biology as a key component of heme, which is vital for several physiological processes. In plants, heme is integral to chlorophyll biosynthesis, where it is involved in the formation of the chlorophyll precursor, protochlorophyllide. Additionally, heme‐containing enzymes, such as cytochrome P450s, are essential for various biochemical pathways, including the synthesis of plant hormones and secondary metabolites that influence growth, stress responses, and disease resistance (Sun et al. 2025). By modulating heme production and function, plants can adapt to environmental conditions and optimize metabolic processes, demonstrating the importance of protoporphyrin IX and its iron‐containing derivative in maintaining plant health and productivity.

It is crucial to note here that future work is needed to further characterize the bioactive metabolites present in both bacterial isolates. Advanced analytical techniques such as high‐resolution mass spectrometry (HRMS), gaschromatography and mass spectrometry (GC–MS), and NMR‐based structure elucidation can be further employed to confirm metabolite identities and assess their biological activity. Additionally, functional assays will help determine their specific roles in plant growth promotion and antifungal activity.

### 4.3 Applied Implications and Prospects

The application of CFSs to crops could result in a myriad of responses, and while the plant growth promotion and biocontrol abilities of such CFSs have been observed practically, a detailed analysis of the host transcriptome and proteome is a necessity. This also poses a key question in agricultural biotechnology: whether disease‐resistant wheat cultivars would benefit from CFS applications. Based on current knowledge in this field, it can be hypothesized that while resistant cultivars possess innate defense mechanisms, CFS treatments could still provide additional benefits such as enhanced vigor, increased nutrient uptake, and stress tolerance (Di Benedetto et al. 2017). It opens an array of possibilities for future studies focusing on investigating whether CFS application further strengthens resistance pathways or enhances overall plant health in these cultivars. The growth‐promoting effects of CFSs, as observed in the current study, suggest that their application may be beneficial even in disease‐free environments. The presence of phytohormones and signaling molecules in CFSs likely contributes to improved root development, biomass accumulation, and grain yield (Marks et al. 2025); therefore, the necessity to study the possible bio‐stimulant properties of CFSs. This suggests that CFSs could function as bio‐stimulants beyond their role in disease suppression, warranting further research into their broader agronomic benefits. It should also be noted that in order to confirm the presence of the named compounds in the CFSs, each compound should be thoroughly identified and purified using reference standards for more accurate verification.

## Conflicts of Interest

The authors declare no conflicts of interest.

## Supporting information


Data S1.

Data S2.

Data S3.

Appendix S4.

## Data Availability

The data that supports the findings of this study are available in the [Link], [Link], [Link], [Link] of this article.
