# Supplementary material for: Leveraging Cell‐Free Supernatants of Phyllospheric Bacteria to Combat Wheat Pathogens and Boost Growth
Source: Plant Environ Interact. 2025 Jul 2;6(4):e70063. doi: 10.1002/pei3.70063 (PMC12222621; doi:10.1002/pei3.70063)
Supplement: Supplementary file 4 — Appendix S4. [file PEI3-6-e70063-s002.docx]

**Supporting Information**

**Supporting Method S1**

A severely diseased leaf from each labeled group was suspended in 250µL of 0.95% saline solution, crushed, and centrifuged at 13,000rpm to separate debris. The supernatant was mixed with 3%CTAB and 0.5% β-mercaptoethanol (100:1) and incubated at 65°C for 25-30 minutes. After centrifugation, the supernatant was mixed with chloroform-isoamyl alcohol (24:1 v/v), and DNA was precipitated with isopropanol. After washing and air-drying, the DNA was suspended in ultrapure water and stored at -20°C. DNA concentrations were checked with a Nanodrop spectrophotometer, and the quality was verified by running samples on a 1% Agarose Gel (Tamari F *et al.,* 2013).

**Table S1:** Colony Morphology of Bacterial Strains

| **16 rRNA gene Sequence (NCBI BLASTn)** | **Accession No.** | **Isolation source** | **Form** | **Margin** | **Elevation** | **Color** | **Surface Appearance** | **Size** |
| --- | --- | --- | --- | --- | --- | --- | --- | --- |
| *Fictibacillus spp.* | MT354437.1 | *Gossypium arboreum*  Leaf episphere | regular | undulate | Flat | Yellow | Dry | large |
| *Serratia marcescens* | MG041387.1 | *Gossypium arboreum*  Leaf endosphere | circular | Entire | Convex | Red | Viscoid | Moderate |

**Table S2:** Test group application details

| **Application Group** | **Application Group Label** | **Application Detail** |
| --- | --- | --- |
| *Serratia marcescens* | *Serratia marcescens* | Wheat plant group applied with Cell Free supernatant of *Serratia marcescens* |
| *Fictibacillus spp.* | *Fictibacillus spp.* | Wheat plant group applied with Cell Free supernatant of *Fictibacillus spp.* |
| Exogenous Salicylic Acid | SA | Wheat plant group applied with Salicylic Acid solution. |
| Exogenous Gibberellic Acid | GA3 | Wheat plant group applied with Gibberellic Acid solution. |
| Negative Control | NC | Wheat plant group applied with sterile distilled water. |

**Table S3:** Grading of powdery mildew disease intensity (Bedika, 2016).

| **Disease Grade** | **Disease Description** |
| --- | --- |
| **0** | No lesions or specks |
| **1** | Small-sized powdery specks infecting less than 1% leaf area |
| **3** | Enlarged irregular powdery growth covering 1-5% leaf area |
| **5** | Powdery growth to form big patches covering 5-25% leaf area |
| **7** | Powdery growth covering 25-50% leaf area followed by yellowing |
| **9** | 100% leaf area covered with powdery growth, yellowing, and dropping of infected leaves |

**Table S4:** PCR profile for 18S rRNA ITS1- ITS2 region amplification

| **Step** | **Temperature °C** | **Duration (min)** | **No. of Cycles** |
| --- | --- | --- | --- |
| Initial Denaturation | 95 | 5 | 1 |
| Denaturation | 95 | 1 | 40 |
| Annealing | 62 | 0.75 |  |
| Extension | 72 | 1 |  |
| Final Extension | 72 | 10 | 1 |
| Hold | 4 | ∞ |  |

**Table S5:** PCR reaction mixture for ITS1-2 gene amplification

**
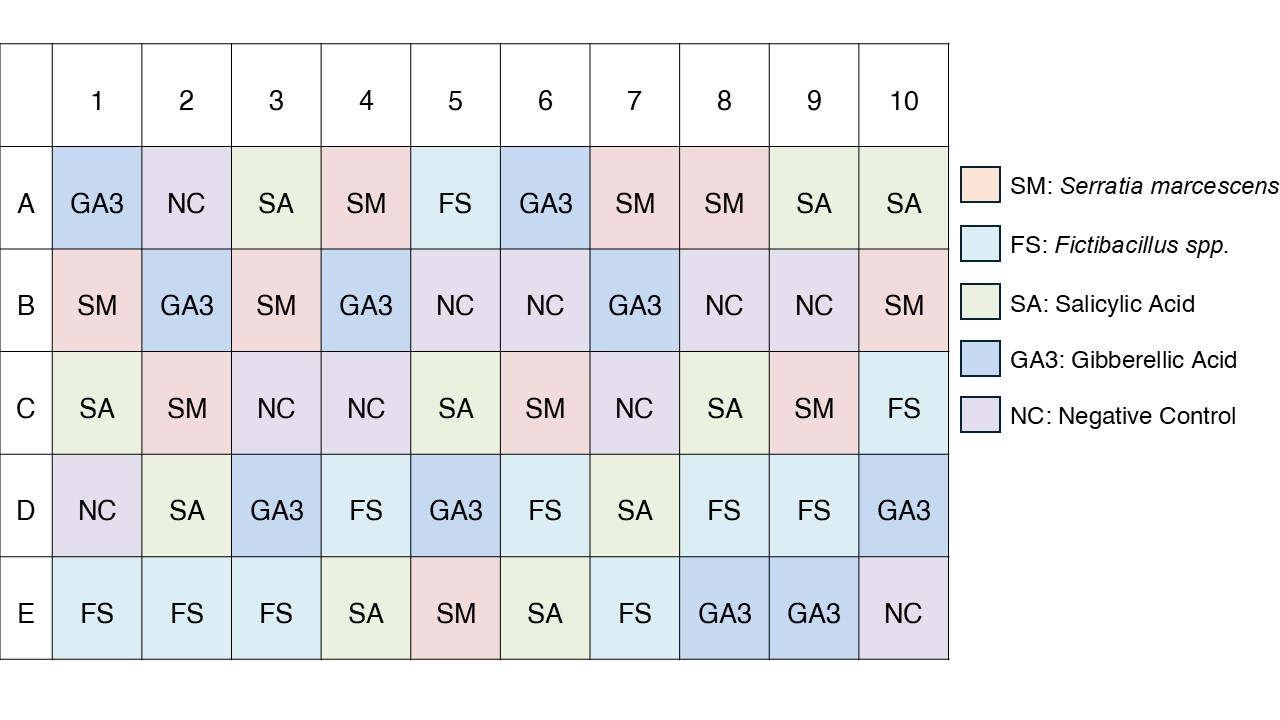
**

| **PCR Contents** | **Quantity/µL** |
| --- | --- |
| PCR Master Mix Dream Taq | 12.5 |
| DMSO | 1.5 |
| MgCl_2_ (2mM) | 1.5 |
| Forward Primer | 1 |
| Reverse Primer | 1 |
| Molecular Grade Water | 5.5 |
| DNA | 2 |
| Total Volume | 25 |

**Figure S1:** Completely randomized block design for the sowing of seeds from all application groups, made using the ‘agricolae’ package in R v4.4.3.


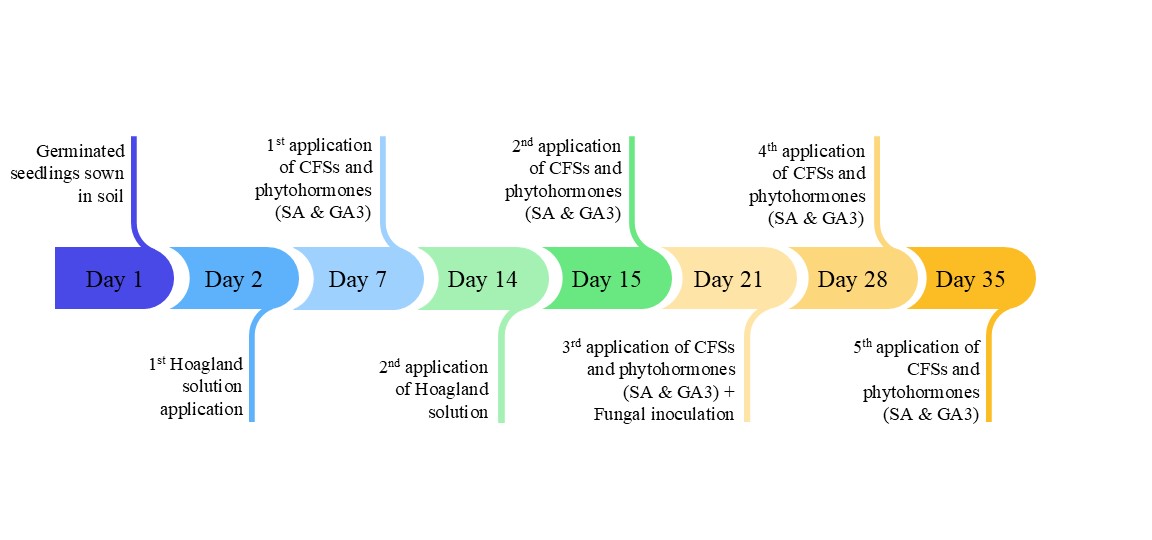
**Figure S2:** Application regime of CFSs of both bacterial strains and phytohormones to the respective test groups, along with experiment timeline.


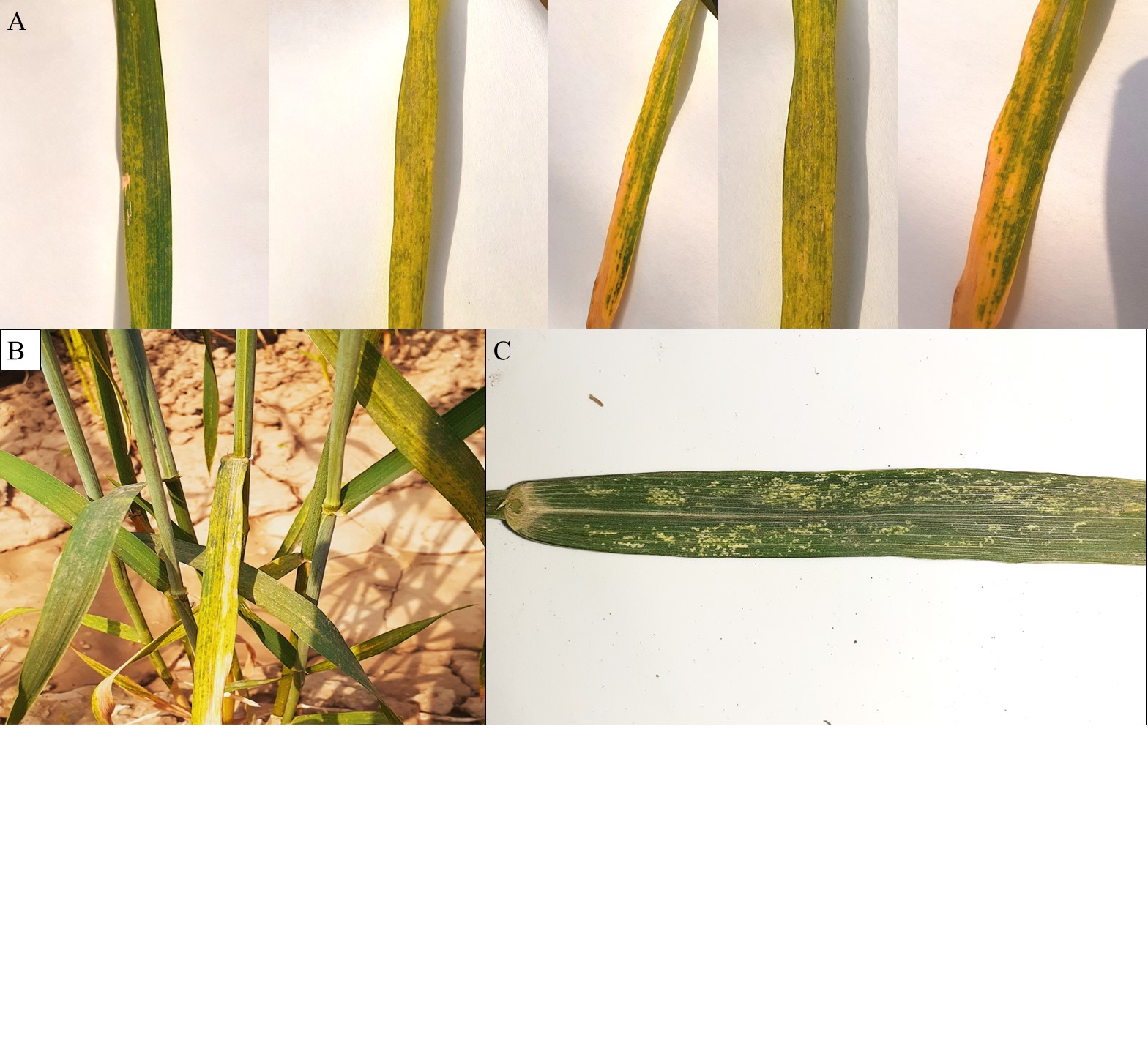


**Figure S3:** Heavily infected wheat plant leaves showing severe symptoms of the two fungal diseases, used for spore suspension preparation for fungal inoculations, **A:** *Puccinia striiformis* infected leaves, **B:** *Puccinia striiformis* infected wheat plant, **C:** *Blumeria graminis* infected leaf shows white powdery characteristic symptoms.

**REFERENCES**

Bedika, M. a. (2016). Screening of Sesame (Sesamum indicum L.) Genotypes for Powdery Mildew Resistance. *National Academy of Agricultural Science (NAAS)*.

Tamari, F., Hinkley, C. S., & Ramprashad, N. (2013). A comparison of DNA extraction methods using Petunia hybrida tissues. *Journal of biomolecular techniques: JBT*, *24*(3), 113.
